# Synergistic effect of ATP for RuvA–RuvB–Holliday junction DNA complex formation

**DOI:** 10.1038/srep18177

**Published:** 2015-12-14

**Authors:** Takuma Iwasa, Yong-Woon Han, Ryo Hiramatsu, Hiroaki Yokota, Kimiko Nakao, Ryuji Yokokawa, Teruo Ono, Yoshie Harada

**Affiliations:** 1 Institute for Integrated Cell-Materials Science (WPI-iCeMS), Kyoto University, Sakyo, Kyoto 606-8501, Japan; 2 Graduate School of Biostudies, Kyoto University, Sakyo, Kyoto 606-8501, Japan; 3 CREST, Japan Science and Technology Corporation (JST), Sanbancho, Chiyoda, Tokyo 102-0075, Japan; 4 Institute for Chemical Research, Kyoto University, Gokasho, Uji, Kyoto 611-0011, Japan; 5 Department of Micro Engineering, Graduate School of Technology, Kyoto University, Sakyo, Kyoto 606-8501, Japan

## Abstract

The *Escherichia coli* RuvB hexameric ring motor proteins, together with RuvAs, promote branch migration of Holliday junction DNA. Zero mode waveguides (ZMWs) constitute of nanosized holes and enable the visualization of a single fluorescent molecule under micromolar order of the molecules, which is applicable to characterize the formation of RuvA–RuvB–Holliday junction DNA complex. In this study, we used ZMWs and counted the number of RuvBs binding to RuvA–Holliday junction DNA complex. Our data demonstrated that different nucleotide analogs increased the amount of Cy5-RuvBs binding to RuvA–Holliday junction DNA complex in the following order: no nucleotide, ADP, ATPγS, and mixture of ADP and ATPγS. These results suggest that not only ATP binding to RuvB but also ATP hydrolysis by RuvB facilitates a stable RuvA–RuvB–Holliday junction DNA complex formation.

Homologous recombination is a crucial biological process not only for the repair of damaged chromosomes, but also for generating genetic diversity. Holliday junction DNA is an important intermediate of the homologous recombination that consists of two homologous duplex DNA molecules linked by a single-stranded crossover. In *Escherichia coli*, RuvA, RuvB, and RuvC are involved in the processing of Holliday junction DNA into mature recombinant DNA molecules^1^,^2^. RuvA is a Holliday junction-specific DNA-binding protein and forms a stable symmetric tetramer^2^,^3^. One RuvA tetramer binds to or two RuvA tetramers sandwich the Holliday junction DNA^3^,^4^. RuvB belongs to the AAA+ ATPase class and functions as a motor protein for branch migration of the Holliday junction^5^. RuvA forms a complex with RuvB which facilitates RuvB binding to DNA^6^. The RuvA–RuvB complex promotes movement of a Holliday junction, known as branch migration^7^,^8^. RuvC is a dimeric endonuclease that cleaves the Holliday junction symmetrically^9^,^10^.

Crystallographic studies showed that *Thermus themophilus* and *Thermatoga maritime* RuvBs have a crescent–like structure with three domains N, M, and C^11^,^12^ (Fig. 1A). Domains N and M are characteristic of the AAA+ ATPase domain with conserved Walker A/B and sensor I/II motifs and are involved in hexamer formation. A unique β-hairpin protruding from domain N physically interacts with RuvA, which is required for RuvA–RuvB complex formation^13^,^14^. Domain C with similar motif to that of the winged helix DNA-binding motif may play a major role in pumping out dsDNA^15^. RuvBs form hexameric rings on dsDNA in the presence of ATP, which sandwich the RuvA tetramer on Holliday junction DNA^11^,^16^. The two rings pump out dsDNA from the junction which results in Holliday junction DNA branch migration^17^. Using the tethered particle method, we and other groups measured RuvA–RuvB mediated Holliday junction DNA branch migration rates and our group also showed that the RuvA–RuvB complex undergoes a rotational movement along the double-helical DNA during Holliday junction DNA branch migration^18^,^19^,^20^,^21^. However, the formation of RuvA–RuvB–Holliday junction DNA complex remains unclear.

The single molecule fluorescence imaging technique using total internal reflection fluorescence (TIRF) microscope is a conventional and powerful method for the characterization of biomolecule interactions in real time^22^,^23^. However, despite of the small detection volume (10^−15^ L), it is very difficult to visualize single fluorescent molecule of interest under submicromolar order concentrations of the fluorescent molecules. To overcome this limitation, zero mode waveguides (ZMWs) have been developed and applied to single molecule real time DNA sequencing^24^,^25^,^26^. ZMWs consist of nanosized holes in an aluminum film that reduces the observational volume to 10^−19^–10^−20^ L. Thus, the ZMWs is said to enable the visualization of a single fluorescent molecule under micromolar order of the molecules^24^. It was also reported that ZMWs were applied to visualize the formation of biomolecules complexes under a micromolar order of fluorescently labeled biomolecules^27^,^28^,^29^,^30^.

In case of RuvB, approximately submicromolar concentrations of RuvB were required for RuvB binding to a Holliday junction DNA. Thus, using TIRF microscope, the observation of fluorescently labeled RuvB binding to a Holliday junction DNA was very difficult. In this study, to characterize RuvB binding to RuvA–Holliday junction DNA complex, we fabricated ZMWs and labeled RuvB with Cy5. Then, we succeeded in visualizing Cy5-RuvBs binding to a RuvA–Holliday junction DNA complex immobilized on the nanohole. We counted the number of Cy5 photobreaching steps under various nucleotide conditions and determined the most probable numbers of RuvBs binding to the complex. Our data shows that, in the presence of ATPγS and ADP, a more stable RuvA–RuvB–Holliday junction complex was formed, suggesting that ATP synergistically facilitates both RuvB hexameric ring formation and RuvA–RuvB–Holliday junction DNA complex formation, which is crucial for Holliday junction DNA branch migration.

## Results

### Labeling RuvB with Cy5

To characterize RuvB binding to Holliday junction DNA using the single molecule fluorescence imaging technique, we constructed and purified a RuvB mutant, RuvB-S39C, and then label the purified RuvB protein with Cy5-maleimide as described in Materials and Methods. We used RuvB-S39C to label RuvB protein by the highly specific conventional reaction between the sulfhydryl group and maleimide group because wild type *E. coli* RuvB has no Cys residues.

**Figure 1:**
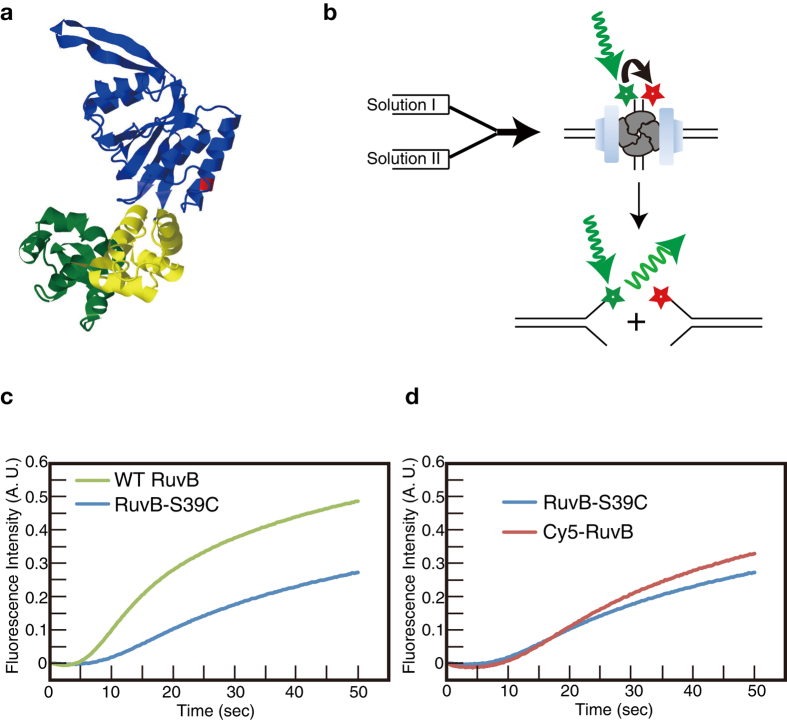
Characterization of Holliday junction DNA branch migration activity RuvB proteins. (**a**) RuvB structure from *T. thermophilus*. Domains N, M, and C are colored blue, yellow, and green, respectively. Gln22 of *T. thermophilus* RuvB corresponding to Ser39 of *E. coli* RuvB is colored red (**b**) Schematic drawing of fluorescence based measurement of RuvA–RuvB mediated Holliday junction DNA branch migration using a stopped-flow system. (**c**) Holliday junction DNA branch migration activities of wild-type RuvB and RuvB-S39C. (**d**) Holliday junction DNA branch migration activity of nonlabeled and Cy5-labeled RuvB-S39C.

To determine the effect of Ser-Cys mutation, we measured branch migration activity of the purified RuvB mutant in the presence of RuvA using a stopped-flow system (Fig. 1). We used fluorescently labeled Holliday junction DNA, which contained a Cy3 fluorophore and a Cy5 fluorophore, at the same end of the DNA (Fig. 1b). Before Holliday junction DNA branch migration took place, Cy3 and Cy5 were located closely to each other and the fluorescence of Cy3 was suppressed by an energy transfer from Cy3 to Cy5. On the other hand, completion of the Holliday junction DNA branch migration yielded separate Cy3-labeled and Cy5-labeled Y-form DNA, and the Cy3 fluorescence resumed (Fig. 1b). As shown in Fig. 1c, fluorescence intensity from Cy3 started increasing from 5 s introduction of wild-type RuvB into the solutions at 25 °C, indicating that Holliday junction DNAs were unwound (Fig. 1c). Our data demonstrates that RuvB-S39C is slightly defective in Holliday junction DNA branch migration activity compared with wild-type RuvB (Fig. 1c). Because RuvB-S39C is still active in Holliday junction DNA branch migration with RuvA, we labeled RuvB-S39C with Cy5 as described in Materials and Methods.

The labeling ratio of Cy5-labeled RuvB-S39C was 42%. The Holliday junction DNA branch migration activity of Cy5-labeled RuvB-S39C was comparable with that of unlabeled RuvB-S39C (Fig. 1d), indicating that the activity was unaffected by Cy5 labeling. Thus, we used Cy5-RuvB-S39C as Cy5-RuvB to characterize RuvB binding to Holliday junction DNA using the single-molecule fluorescence imaging technique.

### ZMW fabrication

**Figure 2:**
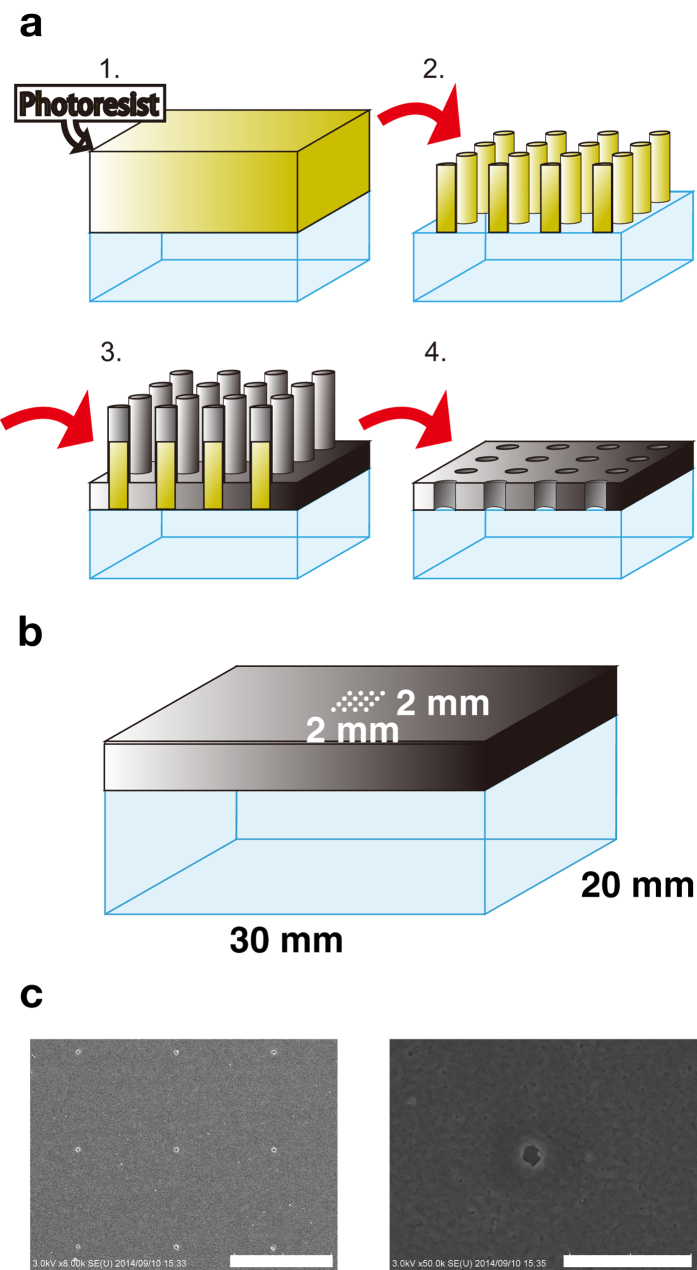
ZMWs used in this study. (**a**) Procedure of ZMW fabrication. (**b**) Design of the ZMWs used in this study. The ZMWs were fabricated at the center of the coverslip, and the fabricated area was 2 × 2 mm. The hole diameter and the distance between the holes were designed as 100 nm and 5 μm, respectively. (**c**) Scanning electron microscopic images of ZMWs. Scale bars indicate 5 μm (left panel) and 1 μm (right panel), respectively.

As described in the methods, 400 nM of Cy5-labeled RuvB-S39C was used to visualize RuvB binding to the RuvA–Holliday junction DNA complex. Because ZMWs enable us to visualize single fluorescently labeled biomolecules at a high concentration of them, we fabricated ZMWs for single molecule Cy5-RuvB observation. Two methods have primarily been reported for ZMW fabrication^26^, the ion-beam milling method^24^ and the metal lift-off method^31^,^32^. In this study, we fabricated ZMWs using the metal lift-off method and obtained nanoscale apertures in aluminum films on the center of fused silica coverslips, as described in Materials and Methods (Fig. 2a,b). After ZMW fabrication, we observed the nanoholes using a scanning electron microscope (SU8000, Hitachi High Technologies) and measured the average diameter of the holes, which was 122 ± 10 nm (Fig. 2c). The hole size was small enough for imaging of Cy5-RuvB binding to a RuvA–Holliday junction DNA complex at the concentration of 400 nM used in this study^24^.

### RuvA–RuvB complexes promote branch migration of Holliday junction DNA immobilized on ZMWs

**Figure 3:**
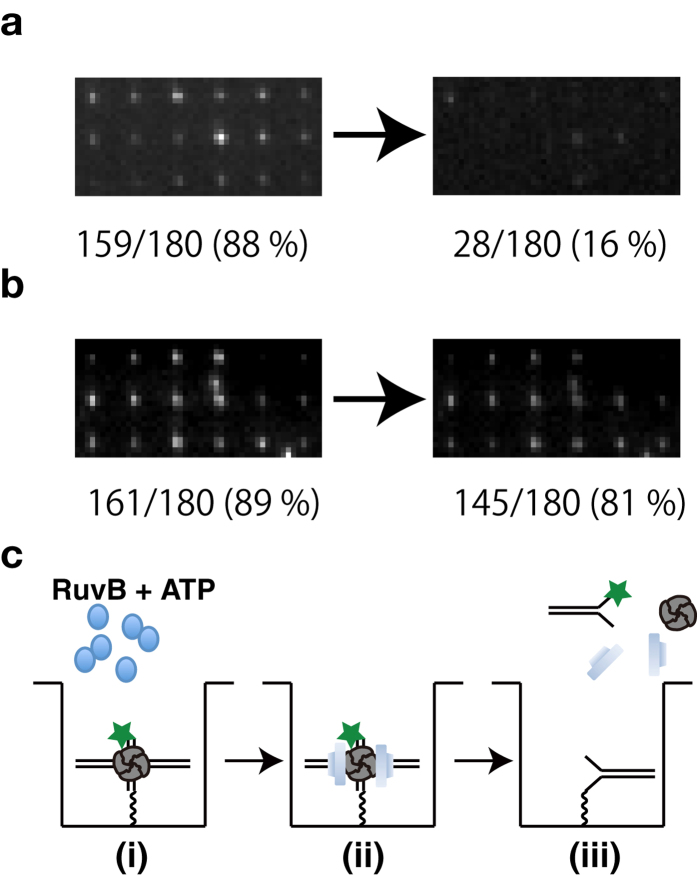
Holliday junction DNA branch migration by RuvA–RuvB complex in the nanoholes. (**a**) Typical fluorescence images from Cy3 labeled Holliday junction DNA immobized on the nanoholes before and after addition of RuvB and ATP. After immobilization of RuvA–Holliday junction DNA complexes in the nanoholes, a mixture containing 400 nM RuvB and 1 mM ATP was added onto the ZMWs. (**b**) Typical fluorescence images from Cy3 labeled Holliday junction DNA immobilized on the nanoholes before and after addition of RuvB alone. After immobilization of RuvA–Holliday junction DNA complexes, mixture containing 400 nM RuvB was added onto the ZMWs. (**c**) Schematic drawing of RuvA–RuvB mediated Holliday junction DNA branch migration in the nanohole. (**i**) Addition of RuvB and ATP to RuvA–Holliday junction DNA complexes immobilized in the nanoholes. (**ii**) RuvA–RuvB–Holliday junction DNA complex formation (**iii**) Dissociation of Cy3-labeled Y-form DNA by RuvA–RuvB mediated Holliday junction DNA branch migration.

To confirm that RuvA–RuvB complexes is capable of promoting branch migration of Holliday junction DNA in the nanoholes, Cy3-labeled Holliday junction DNA was immobilized on a streptavidin coated glass surface, as described in Materials and Methods. The ratio of fluorescent spots of Holliday junction DNA to nanoholes was about 90% before the addition of RuvB proteins to the nanoholes (Fig. 3). After the addition of RuvB proteins with ATP and incubation for 5 min at 25 °C, the ratio of the spots was approximately 16% (Fig. 3a). In contrast, without ATP, the ratio of the spots was almost same as that before the addition of RuvB (Fig. 3b). These results indicate that RuvA–RuvB–Holliday junction DNA complex was formed in the nanohole and that the RuvA–RuvB protein complex with ATP could promote branch migration of Holliday junction DNA immobilized on the nanohole, resulting in dissociation of Cy3-labeled Y-form DNA from the nanohole (Fig. 3c).

### Number of RuvBs binding to RuvA–Holliday junction DNA, immobilized on ZMWs

**Figure 4:**
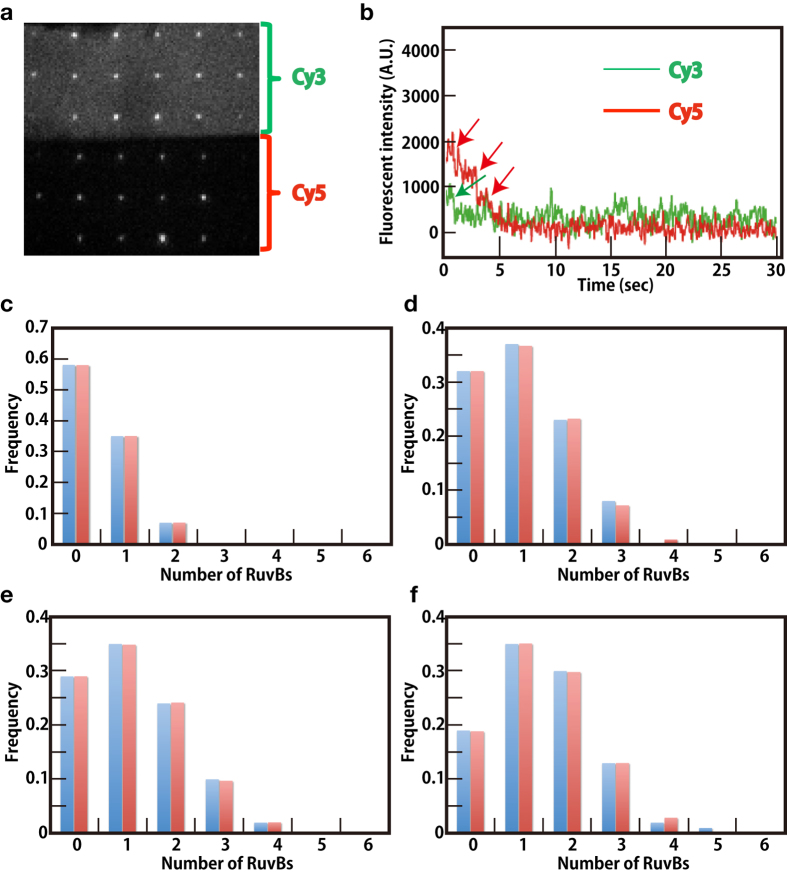
Determination of the number of RuvBs binding to RuvA–Holliday junction DNA complexes. (**a**) Snapshot image of fluorescent spots from Cy3-Holliday junction DNA (upper panel) and Cy5-RuvB (lower panel) in the presence of ATPγS and ADP. (**b**) Representative time trace of Cy3 and Cy5 fluorescence intensity. Each fluorescence intensity decreased in a step manner due to photobreaching. Green and red arrows indicate photobleaching steps of Cy3 and Cy5, respectively. (**c**) Histogram of the number of Cy5-RuvBs binding to a RuvA-Holliday junction DNA in the absence of nucleotide. Blue and red histograms indicate the experimental data and the calculated data, respectively. (**d**) Histogram of the number of Cy5-RuvBs binding to a RuvA–Holliday junction DNA in the presence of 1 mM ADP. (**e**) Histogram of the number of Cy5-RuvBs binding to a RuvA–Holliday junction DNA in the presence of 1 mM ATPγS. (**f**) Histogram of the number of Cy5-RuvBs binding to a RuvA–Holliday junction DNA in the presence of 0.5 mM ADP and 0.5 mM ATPγS.

As described above, we demonstrated that the RuvA–RuvB complex promoted branch migration of Cy3 labeled Holliday junction DNA immobilized on the nanohole, indicating that the RuvA–RuvB–Holliday junction DNA complex was formed in the nanohole. Next, we performed single molecule characterization of the RuvA–RuvB–Holliday junction DNA complex formation using Cy5-labeled RuvB. In the presence of ATP, the RuvA–RuvB protein complex promotes Holliday junction DNA branch migration, resulting in the disassembly of Holliday junction DNA and the formation of Y-form DNA. Here, we used ATPγS and ADP as nucleotide cofactors. It was impossible for us to visualize Cy5-RuvB binding to the junction in the presence of ATP. We observed bright spots that emitted stable Cy3 and Cy5 fluorescence from nanoholes on which Cy3-Holliday junction DNA was immobilized. Most of the Cy3 and Cy5 fluorescence intensity decreased in a stepwise manner due to photobreaching (Fig. 4a,b, Supplement Movie 1). The numbers of photobreaching steps corresponded with the number of Cy3-Holliday junction DNA immobilized on the nanohole and Cy5-RuvB binding to the RuvA–Holliday junction DNA complexes, respectively. The mean signal-to-noise ratios of Cy3 and Cy5 were 2.2 and 3.9, respectively. As shown in Fig. 4b, we focused on nanoholes containing single Cy3-Holliday junction DNA and counted the number of photobleaching steps from Cy5-RuvB to determine the number of Cy5-RuvBs binding to the complex in the nanoholes containing single Holliday junction DNA.

We characterized RuvB binding to the complex under conditions without nucleotides, with ADP, ATPγS or both ADP and ATPγS (Fig. 4c–f). Because the labeling ratio of Cy5-RuvB was 42%, the number of photobleaching step did not represent the number of RuvB. Thus, to determine the number of RuvBs binding to the complex, we fitted our experimental data with calculated data. As shown in Fig. 1d, our data showed that branch migration activity of Cy5-RuvB was comparable to that of RuvB-S39C. Thus, we regarded affinities to the RuvA–Holliday junction DNA complex of Cy5-RuvB and RuvB-S39C as almost equivalent. Previous biochemical assays indicated that in the absence of nucleotide and divalent cations such as Mg^2+^, RuvBs exist as a monomer and/or dimer^2^,^33^,^34^. We considered the RuvB protomer as a monomer and calculated a binominal distribution between 42% of Cy5-RuvB and 58% of nonlabeled RuvB to obtain the calculated data (Supplementary Table S1). A least-squares fitting technique was performed, and we determined the minima of the sum of the square residuals calculated using least-squares fitting technique between our experimental data and the calculated data to fit our experimental data with the calculated data (Fig. 4c–f and Table 1). The fitted results indicate that in the absence of ADP or ATPγS, 77% of Holliday junction DNA interacted with RuvB, and that 37% and 40% of the Holliday junction DNA had one or two RuvBs, respectively. In contrast, the results indicate that in the presence of ADP, 90% of Holliday junction DNAs interacted with RuvB, and 30% and 29% of the Holliday junction DNA had three or four RuvBs, respectively. These results indicate that the presence of nucleotide promotes more RuvBs binding to a RuvA–Holliday junction DNA complex. The fitted results also indicate that in the presence of ATPγS, 92% of Holliday junction DNA interacted with RuvBs, and 31% and 12% of the Holliday junction DNA interacted with four or five RuvBs, respectively. This indicates that ATPγS promotes more RuvBs binding to a RuvA–Holliday junction DNA complex than ADP. Intriguingly, the fitted results indicate that in the presence of ADP and ATPγS, 98% of Holliday junction DNA interacted with RuvBs and 31%, 37%, 14%, and 3% of the Holliday junction DNA had three, four, five, and six RuvBs, respectively (Table 1).

In the case that the RuvB protomer is a dimer, we considered that the distribution of Cy5-RuvB in dimer was random, and as shown in Supplementary Table S2, similar calculated data was obtained and compared with that based on the RuvB monomer model. We fitted our data with the calculated data to obtain the distribution of the number of RuvBs binding to a RuvA–Holliday junction DNA complex (Table 2). Even though the numbers of RuvBs binding to a RuvA–Holliday junction DNA complex were only even numbers, the fitted data was almost similar to that from the RuvB monomer model.

## Discussion

To date, RuvB properties on RuvA–RuvB–Holliday junction complex formation or DNA–binding activity have been largely characterized by an electrophoresis mobility shift assay (EMSA) with glutaraldehyde cross-linking^35^,^36^,^37^, because of the weak stability of the RuvB–DNA complex. As reported previously, our EMSA data showed that the RuvA–Holliday junction DNA complex formed complexes with RuvB in the presence of ATPγS^13^. However, we could not measure the number of RuvBs in the complex.

Single fluorescence imaging techniques enabled us to characterize the protein–protein or protein–DNA complex in more detail. We could visualize the assembly or disassembly processes of the complex and count the number of molecules constituting the complex in real time. In this study, to characterize the single molecule formation of RuvA–RuvB–Holliday junction DNA complex, we labeled RuvB with Cy5 and fabricated ZMWs. We measured the number of RuvBs binding to a RuvA–Holliday junction DNA complex under various nucleotide conditions (Fig. 4). Interestingly, our results indicate that in the absence of ATPγS or ADP, RuvBs formed complexes with RuvA–Holliday junction DNA complexes and all of the complexes contained one or two RuvBs. Our results also indicate that in the presence of ATPγS or ADP, about 90% of Holliday junction DNA formed complexes with RuvBs. To date, the crystallographic RuvA–RuvB complex structure containing AMPPNP or ADP has been resolved; however, structural information of the RuvA–RuvB complex without a nucleotide has not been reported^14^. These results indicate that in the absence of ATP, the complex is less stable than it is with ATP. Previous biochemical and structural analyses of RuvA and RuvB suggest that the C-terminal domain (domain III) of RuvA and the β-hairpin protruding from domain N of RuvB is responsible for the RuvA–RuvB interaction and in the absence of ATP or ADP, RuvA and RuvB form the complex containing the RuvA tetramer and RuvB dimer^13^,^37^,^38^,^39^. However, electron microscopic observation of RuvA–RuvB–Holliday junction DNA showed that the β-hairpins were located at the top of RuvB hexameric ring and faced to domain III of the RuvA tetramer bound to Holliday junction DNA^13^,^14^,^16^. Previously, the crystallographic RuvA domain III–RuvB structure revealed that the β-hairpin was partly involved in the interface of RuvB subunits assembly^14^. The interface contains an arginine finger, which senses ATP hydrolysis in the adjacent RuvB subunit^40^. The arginine finger is located between Sensor I and Sensor II^5^, which are also involved in ATP binding and hydrolysis in cooperation with Walker A and B motifs. Our data suggests that ATP or ADP binding to RuvB induces structural changes, not only for a higher oligomeric formation of RuvB, but also for a stable RuvA–RuvB interaction.

**Figure 5:**
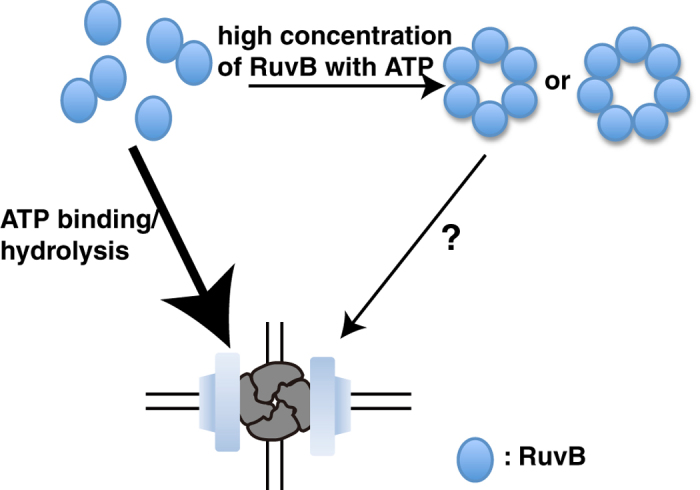
DNA loading model of RuvB.

In the presence of ATPγS and ADP, 97%–98% of Holliday junction DNAs interacted with RuvBs and approximately 3% of the complexes contained six RuvBs (Table 1 and 2). This demonstrated that different nucleotide analogs increased the number of RuvBs binding to RuvA–Holliday junction DNA in the following order: no nucleotide, ADP, ATPγS, and both of ADP and ATPγS. Because ATPγS is ATP nonhydrolyzable analogue, our data suggested that RuvB hexamer containing ATP and ADP was a more stable complex compared with other RuvB hexamers. Like F1-ATPase, RuvB hexamer constituting one pair each of ATP-bound, ADP-bound, and nucleotide-free monomers is supposed to be a stable RuvB hexamer^40^,^41^,^42^. Our data also showed that in the presence of ATPγS and ADP, most of RuvA–RuvB–Holliday junction DNA complexes contained three, four, or five RuvBs at 400 nM of the Cy5-RuvB. These complexes might indicate the intermediate RuvB hexameric ring formation, suggesting that RuvB monomers and/or dimers assemble on the RuvA–Holliday junction complex in the presence of ATP to form RuvA–RuvB–Holliday junction DNA complex at a low concentration of RuvB (Fig. 5)^43^. Electron microscopic imaging of RuvA–RuvB–Holliday junction DNA complexes showed that RuvBs formed a hexameric ring on dsDNA in the presence of ATPγS, suggesting that ATP hydrolysis was not required for hexameric ring formation. However, our data demonstrated that in the presence of ATPγS and ADP, more RuvBs interacted with RuvA–Holliday junction DNA complexes, compared with that in the presence of ATPγS only. Furthermore, the stopped flow analysis demonstrated that RuvA–RuvB mediated Holliday junction DNA branch migration started several seconds after mixing RuvA, RuvB, Holliday junction DNA, and ATP (Fig. 1), indicating that hexameric RuvB rings formed on Holliday junction DNA in several seconds. However, as shown in Fig. 4d, less RuvB hexameric rings formed on Holliday junction at 400 nM of RuvB in the presence of ATPγS. These data indicate that the rate constant of RuvB hexameric ring formation on a RuvA–Holliday junction DNA complex in the presence of ATP was much faster than that in the presence of ATPγS. These results suggest that not only ATP binding to RuvBs but also ATP hydrolysis by RuvBs facilitated RuvB hexameric ring formation on dsDNA.

The RuvB protomer was recently supposed to be dimer^13^; however, we could not rule out the possibility that RuvB exits as a monomer at low concentration of RuvB in the absence of ATP and Mg^2+^. Thus, in this study, we determined the distribution of the number of RuvBs binding to a RuvA–Holliday junction DNA based on two models. One model is based on the model that the RuvB protomer is a monomer (Table 1), and another model assumes that the RuvB protomer is a dimer and we considered that Cy5-RuvB existed in RuvB dimers at random (Table 2). These data were comparable with each other, even though in the case of RuvB dimer model, only even numbers of RuvBs binds to a RuvA–Holliday junction DNA complex. To characterize the RuvB loading process onto Holliday junction DNA in more detail, we need to visualize the initial steps of RuvB loading to DNA in the presence of ATP. We are currently customizing our ZMWs combining with microfluidic system as reported previously^23^,^44^,^45^, which enables us to visualize the initial step of the complex formation process in real time. Not only the customizing system, but also higher labeling ratio of fluorescently labeled RuvB are required; however, in this study, the labeling ratio was 42%. In this study, we constructed two Ser-Cys mutant, RuvB-S39C and RuvB-S9C. RuvB-S9C was defective in Holliday junction DNA branch migration activity and we did not use the RuvB mutant protein (data not shown). However, *E. coli* RuvB has 11 Ser residues, and we are now constructing other Ser-Cys mutant to obtain fluorescently labeled RuvB with high labeling ratio.

As described above, the labeling ratio of RuvB-S39C with Cy5 was 42%. In this study, to determine the number of RuvBs binding to a RuvA-Holliday junction DNA, we considered two possibilities. One possibility is that the RuvB protomer is a monomer (Table 1) and another possibility is that the RuvB protomer is a dimer and Cy5-RuvBs are distributed throughout RuvB dimer at random (Table 2). Furthermore, we assumed that RuvB stably forms a dimer and only one RuvB in the dimer can be labeled by Cy5. In this case, all of Cy5 labeled RuvB dimers contain a Cy5-RuvB and a non-labeled RuvB. Even though we do not have any data to support this model, we calculated the binominal distribution based on this assumption. As shown in Supplementary Table S3, we assumed that 84% of RuvB dimers contained a Cy5-RuvB and 16% of RuvB dimers were non labeled RuvB dimers. We also calculated the binominal distribution between 84% of Cy5 labeled RuvB dimers and 16% of non labeled RuvB dimers to obtain the calculated data (Supplementary Table 3). We fitted our data with the calculated data to obtain the distribution of the number of RuvBs binding to a RuvA–Holliday junction DNA complex (Table 3). Compared with the data from Table 1 and 2, the number of RuvBs binding to a RuvA–Holliday junction DNA complex increased by approximately two-fold; however, the number of RuvBs binding to the complex was 10 at the maximum. These data suggested that more RuvB was required for the formation of double RuvB hexameric rings with a RuvA–Holliday junction DNA complex in the presence of ADP and ATPγS. To visualize RuvBs binding to a RuvA–Holliday junction DNA complex at 10 μM RuvB using ZMWs, the diameter of the nanoholes should be narrow and approximately 50 nm.

Previous biochemical analyses and electron microscopic observations demonstrated that RuvBs form a hexameric or heptameric ring in solution with ATP or ATPγS, suggesting that the RuvB rings directly load onto Holliday junction DNA, resulting in a RuvA–RuvB–Holliday junction DNA complex formation at a high concentration of RuvB^46^. However, the reaction mechanism as to how the RuvB rings directly load onto dsDNA has not yet been clarified (Fig. 5). As discussed above, we are now customizing and improving our ZMWs and further analysis of the formation of RuvA–RuvB–Holliday junction DNA complex is now in progress.

## Materials and Methods

### Bacterial strain and Plasmids

*E. coli* HRS4000 (BL21 (DE3)-Δ*ruvABC*100::*kan*) was used for protein overexpression^13^. The expression plasmids pAF134 and pRB100 were used for wild type RuvA and RuvB expression, respectively^13^.

### Site-directed mutagenesis

The expression plasmid for RuvB-S39C was constructed by PCR mediated site-directed mutagenesis as described previously^40^. Two oligonucleotides, S39C-F and S39C-R were used for construction of the plasmid for RuvB-S39C. The sequences of each oligonucleotide were as below. S39C-F; 5′-CAGCCGCAGGTTCGTTGCCAGATGGAGATTTTC-3′. S39C-R; 5′-GAAAATCTCCATCTGGCAACGAACCTGCGGCTG-3′.

### Protein purification and Cy5-RuvB preparation

RuvA and RuvB proteins were purified as previously described^3^,^21^.

Using RuvB-S39C and Cy5-maleimide, Cy5-RuvB was prepared as below. Approximately 1.0 mg of Cy5-maleimide was dissolved in 10 μL of *N*, *N*-dimethylformamide (DMF). RuvB-S39C and Cy5-maleimide was mixed at the ratio of 1:5 in 1.0 mL of mixture containing 10 μM RuvB-S39C, 50 μM Cy5-maleimide, 20 mM HEPES-KOH (pH 7.0), and 8% Glycerol. The mixture was incubated for 16 h at 4 °C. After the coupling reaction, purification of Cy5-RuvB was performed using Resource Q (GE) in a purification buffer containing 30 mM Tris-HCl (pH 7.5), 1 mM EDTA and 15% Glycerol. The protein was eluted with a 20 mL linear gradient from 0 M to 1 M NaCl in a purification buffer.

### Holliday junction DNA preparation

Two Holliday junction DNAs were prepared as below. Four oligonucleotides (JY21-Cy5, JY22-Cy3, JY23, and JY24) were mixed in a buffer containing 50 mM Tris-HCl (pH 7.5), 100 mM NaCl, and 10 mM MgCl_2_, and Holliday junction DNA was constructed as described previously and used for the branch migration assay^37^. Another Holliday junction DNA was constructed by annealing four oligonucleotides (JY21, JY22-Cy3, JY23-L, and JY24-L) and used in the ZMW analysis. The sequences of each oligonucleotide were as follows. JY21; 5′-CGAGCGACAGGAACCTCGAGAAGCTTCAATCGGCTCAGACCGAGCAGAATTC-3′. JY22; 5′-GAATTCTGCTCGGTCTCTCGGCAGATCTCGAGAATCGACGCTAGCAAGTGAC-3′. JY23; 5′-GTCACTTGCTAGCGTCGATTCTCGAGATCTGCCGAGACTGGCTGTGGGATCC-3′. JY23-L; 5′-GTCACTTGCTAGCGTCGATTCTCGAGATCTGCCGAGACTGGCTGTGGGATCCGAGCTGTCTAGAGACATCGA-3′. JY24; 5′-GGATCCCACAGCCAGTGAGCCGATTGAAGCTTCTCGAGGTTCCTGTCGCTCG-3′. JY24-L; 5′-TTTTTTTTTTTTTTTTTTTTTCGATGTCTCTAGACAGCTCGGATCCCACAGCCAGTGAGCCGATTGAAGCTTCTCGAGGTTCCTGTCGCTCG-3′. The sequence of JY21-Cy5 was same as JY21 but contained Cy5 at the 3′ end. The sequence of JY22-Cy3 was same as JY22 but contained Cy3 at the 5′ end. JY24-L contained biotin at 5′ end.

### Branch migration assay

Branch migration activity of the RuvA–RuvB complex was carried out using a stopped-flow spectrafluorometer (Model SX20; Applied Photophysics) equipped with a photomultiplier tube. The filter used with the photomultiplier was Semrock FF01-567/15-25. Excitation was at 520 nm for Cy3. Solution I and Solution II were prepared as below. Solution I contained 100 nM RuvA, 10 nM Holliday junction DNA, 20 mM Tris-HCl (pH 8.0), 10 mM MgCl_2_ and 1 mM DTT. Solution II contained 400 nM RuvB, 2 mM ATP, 20 mM Tris-HCl (pH 8.0), 10 mM MgCl_2_ and 1 mM DTT. Branch migration starts by mixing Solution I and Solution II at 25 °C. All data curves represent the average of at least four experiments.

### ZMW fabrication

Fused silica coverslips were immersed in 4% ammonium and 4.3% hydrogen peroxide for 10 min at 75 °C and then washed thoroughly with deionized water. The coverslips were dried with an air blower, and then baked at 200 °C for 10 min. The coverslips were then cleaned by air plasma at 18 W for 10 min. Before coating with Hexamethyldisilazane (HMDS, AZ Electronic Materials), the coverslips were immersed in 2-butanone and cleaned by sonication for 5 min. A resist film of ma-N 2403 (Micro resist technology) was then coated on the HMDS coated coverslip with a spin coater. The ESPACER (Showa Denko) was then coated on the ma-N 2403 coated coverslip. Electron beam (EB) lithography (Elionix Inc.) was performed with an accelerating voltage of 80 kV, and a beam current of 100 pA. After EB patterning, the coverslips were immersed in deionized water for 30 s, and the pattern was then developed by immersing it in ma-D 525 (Micro resist technology) for 2 min. After development, the coverslips were washed thoroughly with deionized water for 5 min and dried with an air blower. Aluminum coating was performed in a thermal evaporator using a BN composite boat. The thickness of the coated aluminum was monitored using a thickness monitor (Eiko Engineering Co. Ltd.). After aluminum coating, the remaining photoresist was lifted off by immersing it in 2-butanone with sonication for 5 min.

The ZMWs were observed by scanning electron microscopy (SU-8000; Hitachi) and the diameter of each nanohole was measured.

### Microscope

Samples were observed at 25 ± 2 °C on an Olympus IX71 inverted microscope with a 100X oil-immersion objective as described previously^47^. An Nd:YAG laser (Compass 315M, Coherent) and a HeNe laser (05-LHP-991, Melles Griot) were used to excite Cy3 at 532 nm and Cy5 at 633 nm, respectively. The fluorescent signals from the samples were passed through dichroic mirrors to separate the fluorescences of Cy3 and Cy5. Barrier filters (580DF30 for Cy3 and 670DF40 for Cy5) were used to eliminate the background light. The filtered fluorescence signals (565–595 nm for Cy3 and 650–690 nm for Cy5) were imaged using a dual view apparatus and recorded with a high-sensitivity CCD camera. The recorded images were analyzed using Image Pro Plus.

### Polyethylene glycol coating

ZMWs were washed with acetone under sonication for 5 min and then washed with 2-propanol under sonication for 5 min. The ZMWs were dried with an air blower and cleaned with air plasma at 18 W for 5 min. The ZMWs were immersed in a preheated 0.6% (vol/vol) aqueous solution of poly(vinylphosphonic acid) (Funakoshi) for 10 min at 90 °C. They were washed briefly with deionized water, dried with an air blower, and annealed on a hot plate at 80 °C for 10 min. The Polyethylene glycol (PEG) coating was then performed as described previously^22^. The ZMWs were amine modified with 2% (vol/vol) of N-2-(aminoethyl)-3-aminopropyl-triethoxysilane (KBE-603, Shin-Etsu Chemical, Japan) in stirred methanol containing 135 mM acetic acid and 4% (vol/vol) MilliQ water for 20 min at room temperature. The amino modified ZMWs were washed with MilliQ water and dried on a clean bench with an air blower. The dried ZMWs were coated with PEG for 3 h at room temperature with a drop containing 10 mg (50 μl of 200 mg/ml) *N*-hydroxy-succinimidyl (NHS) group (SUNBRIGHT ME-50CS, M.W. = 5,000 Da, NOF Corporation, Japan) and 0.1 mg NHS-Bio-PEG (13 5000-25-35, M.W. = 5,000 Da, Rapp Polymere, Tuebingen, Germany) dissolved in 50 mM MOPS buffer (pH 7.5). After the coating, the ZMWs were washed with MilliQ water and dried on a clean bench with an air blower.

### Single-molecule imaging analysis

To observe RuvB binding to RuvA–Hollliday junction DNAs, the DNA was immobilized in the nanoholes. PEG-coated ZMWs were incubated for 5 min at room temperature with a drop containing 1% F127 in Water. The ZMWs were then rinsed with Buffer A (20 mM HEPES-KOH (pH 8.0), 10 mM MgCl_2_). PEG-coated ZMWs were incubated for 5 min at room temperature with a drop containing 0.06 mg/ml streptavidin in Buffer A. The ZMWs were then rinsed with Buffer A. A 20 μl mixture containing 400 nM RuvA and 40 nM Holliday junction DNA in Buffer A was added to the ZMWs and incubated for 5 min. The ZMWs were rinced with Buffer A containing 2.5 mM Protocatechuic acid (PCA, Sigma), 250 nM Protocatechuate-3,4-dioxygenase (PCD, Sigma), and 2 mM Trolox (Sigma) to wash out the unbound RuvA and Holliday junction DNA. The ZMW was set on the microscope. A mixture containing 400 nM Cy5-RuvBs and the indicated amount of nucleotides in Buffer A with 2.5 mM PCA, 250 nM PCD, and 2 mM Trolox was added to the ZMW and the fluorescence imaging from Cy3 labeled Holliday junction DNA and Cy5-RuvB were recorded.

### Data fitting

The calculated data were obtained by a binominal distribution between 42% of Cy5-RuvB and 58% of nonlabeled RuvB as shown in Supplementary Table S1. The sums of the square residuals between our experimental data and the calculated data with no nucleotides, with ADP, ATPγS, and both ADP and ATPγS were depicted as F_no-nucleotide_, F_ADP_, F_ATP_γS, and F_ADP_ and ATPγS, respectively.

F_no-nucleotide_ = (a/100 + 0.58 × b/100 + 0.3364 × c/100 + 0.1551 × d/100 + 0.1132 × e/100 + 0.0656 × f/100 + 0.0381 × g/100 − 0.58)^2^ + (0.42 × b/100 + 0.4872 × c/100 + 0.4239 × d/100 + 0.3278 × e/100 + 0.2377 × f/100 + 0.1654 × g/100 − 0.35)^2^ + (0.1764 × c/100 + 0.3069 × d/100 + 0.3560 × e/100 + 0.3442 × f/100 + 0.2994 × g/100 − 0.07)^2^ + (0.0741 × d/100 + 0.1719 × e/100 + 0.2492 × f/100 + 0.2891 × g/100)^2^ + (0.0311 × e/100 + 0.0902 × f/100 + 0.1570 × g/100)^2^ + (0.0131 × f/100 + 0.0455 × g/100)^2^ + (0.0055 × g/100)^2^.

F_ADP_ = (a/100 + 0.58 × b/100 + 0.3364 × c/100 + 0.1551 × d/100 + 0.1132 × e/100 + 0.0656 × f/100 + 0.0381 × g/100 − 0.32)^2^ + (0.42 × b/100 + 0.4872 × c/100 + 0.4239 × d/100 + 0.3278 × e/100 + 0.2377 × f/100 + 0.1654 × g/100 − 0.37)^2^ + (0.1764 × c/100 + 0.3069 × d/100 + 0.3560 × e/100 + 0.3442 × f/100 + 0.2994 × g/100 − 0.23)^2^ + (0.0741 × d/100 + 0.1719 × e/100 + 0.2492 × f/100 + 0.2891 × g/100  − 0.08)^2^ + (0.0311 × e/100 + 0.0902 × f/100 + 0.1570 × g/100)^2^ + (0.0131 × f/100 + 0.0455 × g/100)^2^ + (0.0055 × g/100)^2^.

F_ATP_γ_S_ = (a/100 + 0.58 × b/100 + 0.3364 × c/100 + 0.1551 × d/100 + 0.1132 × e/100 + 0.0656 × f/100 + 0.0381 × g/100 − 0.29)^2^ + (0.42 × b/100 + 0.4872 × c/100 + 0.4239 × d/100 + 0.3278 × e/100 + 0.2377 × f/100 + 0.1654 × g/100 − 0.35)^2^ + (0.1764 × c/100 + 0.3069 × d/100 + 0.3560 × e/100 + 0.3442 × f/100 + 0.2994 × g/100 − 0.24)^2^ + (0.0741 × d/100 + 0.1719 × e/100 + 0.2492 × f/100 + 0.2891 × g/100 − 0.10)^2^ + (0.0311 × e/100 + 0.0902 × f/100 + 0.1570 × g/100 − 0.02)^2^ + (0.0131 × f/100 + 0.0455 × g/100)^2^ + (0.0055 × g/100)^2^.

F_ADP and ATP_γ_S_ = (a/100 + 0.58 × b/100 + 0.3364 × c/100 + 0.1551 × d/100 + 0.1132 × e/100 + 0.0656 × f/100 + 0.0381 × g/100 − 0.19)^2^ + (0.42 × b/100 + 0.4872 × c/100 + 0.4239 × d/100 + 0.3278 × e/100 + 0.2377 × f/100 + 0.1654 × g/100 − 0.35)^2^ + (0.1764 × c/100 + 0.3069 × d/100 + 0.3560 × e/100 + 0.3442 × f/100 + 0.2994 × g/100 − 0.30)^2^ + (0.0741 × d/100 + 0.1719 × e/100 + 0.2492 × f/100 + 0.2891 × g/100 − 0.13)^2^ + (0.0311 × e/100 + 0.0902 × f/100 + 0.1570 × g/100 − 0.02)^2^ + (0.0131 × f/100 + 0.0455 × g/100 − 0.01)^2^ + (0.0055 × g/100)^2^.

a, b, c, d, e, f, and g indicate percentages of 0, 1, 2, 3, 4, 5, and 6 RuvBs binding to a RuvA-Holliday junction DNA. The a, b, c, d, e, f, and g satisfied the following conditions.


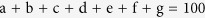


a, b, c, d, e, f, and g were nonnegative integers.

The inequalities as below were not allowed.


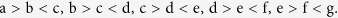


In case that the RuvB protomer is a dimer containing Cy5-RuvB at random, the calculated data were also obtained by a binominal distribution between 42% of Cy5-RuvB and 58% of nonlabeled RuvB as shown in Supplementary Table S2. Compared with the data from Supplementary Table S1, binominal distribution of 1, 3, or 5 RuvBs were excluded. The sums of the square residuals between our experimental data and the calculated data with no nucleotides, with ADP, ATPγS, and both ADP and ATPγS were depicted as G_no-nucleotide_, G_ADP_, G_ATP_γ_S_, and G_ADP and ATP_γ_S_, respectively.

G_no-nucleotide_ = (h/100 + 0.3364 × i/100 + 0.1132 × j/100 + 0.0381 × k/100 − 0.58)^2^ + (0.4872 × i/100 + 0.3278 × j/100 + 0.1654 × k/100 − 0.35)^2^ + (0.1764 × i/100 + 0.3560 × j/100 + 0.2994 × k/100 − 0.07)^2^ + (0.1719 × j/100 + 0.2891 × k/100)^2^ + (0.0311 × j/100 + 0.1570 × k/100)^2^ + (0.0455 × k/100)^2^ + (0.0055 × k/100)^2^.

G_ADP_ = (h/100 + 0.3364 × i/100 + 0.1132 × j/100 + 0.0381 × k/100  − 0.32)^2^ + (0.4872 × i/100 + 0.3278 × j/100 + 0.1654 × k/100 − 0.37)^2^ + (0.1764 × i/100 + 0.3560 × j/100 + 0.2994 × k/100  − 0.23)^2^ + (0.1719 × j/100 + 0.2891 × k/100  − 0.08)^2^ + (0.0311 × j/100 + 0.1570 × k/100)^2^ + (0.0455 × k/100)^2^ + (0.0055 × k/100)^2^.

G_ATP_γ_S_ = (h/100 + 0.3364 × i/100 + 0.1132 × j/100 + 0.0381 × k/100 − 0.29)^2^ + (0.4872 × i/100 + 0.3278 × j/100 + 0.1654 × k/100 − 0.35)^2^ + (0.1764 × i/100 + 0.3560 × j/100 + 0.2994 × k/100 − 0.24)^2^ + (0.1719 × j/100 + 0.2891 × k/100 − 0.10)^2^ + (0.0311 × j/100 + 0.1570 × k/100 – 0.02)^2^ + (0.0455 × k/100)^2^ + (0.0055 × k/100)^2^.

G_ADP and ATP_γ_S_ = (h/100 + 0.3364 × i/100 + 0.1132 × j/100 + 0.0381 × k/100 − 0.19)^2^ + (0.4872 × i/100 + 0.3278 × j/100 + 0.1654 × k/100 − 0.35)^2^ + (0.1764 × i/100 + 0.3560 × j/100 + 0.2994 × k/100 − 0.30)^2^ + (0.1719 × j/100 + 0.2891 × k/100 − 0.13)^2^ + (0.0311 × j/100 + 0.1570 × k/100 – 0.02)^2^ + (0.0455 × k/100 – 0.01)^2^ + (0.0055 × k/100)^2^.

h, i, j, and k indicate percentages of 0, 2, 4, and 6 RuvB dimers binding to a RuvA-Holliday junction DNA. The h, i, j, and k satisfied the following conditions.


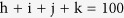


h, i, j, and k were nonnegative integers.

The inequalities as below were not allowed.


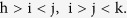


In case that all of Cy5 labeled RuvB dimers contain a Cy5-RuvB and a non-labeled RuvB, the calculated data were obtained by a binominal distribution between 84% of RuvB dimer containing single Cy5-RuvB and 16% of nonlabeled RuvB dimer as shown in Supplemental Table S3. The sums of the square residuals between our experimental data and the calculated data with no nucleotides, with ADP, ATPγS, and both ADP and ATPγS were depicted as H_no-nucleotide_, H_ADP_, H_ATPγS_, and H_ADP and ATPγS_, respectively.

H_no-nucleotide_ = (l/100 + 0.16 × m/100 + 0.0256 × n/100 + 0.0041 × p/100 + 0.0007 × q/100 + 0.0001 × r/100 − 0.58)^2^ + (0.84 × m/100 + 0.2688 × n/100 + 0.0645 × p/100 + 0.0138 × q/100 + 0.0028 × r/100 + 0.0005 × s/100 − 0.35)^2^ + (0.7056 × n/100 + 0.3387 × p/100 + 0.1084 × q/100 + 0.0289 × r/100 + 0.0069 × s/100 − 0.07)^2^ + (0.5927 × p/100 + 0.3793 × q/100 + 0.1517 × r/100 + 0.0486 × s/100)^2^ + (0.4978 × q/100 + 0.3983 × r/100 + 0.1912 × s/100)^2^ + (0.4182 × r/100 + 0.4015 × s/100)^2^ + (0.3513 × s/100)^2^.

H_ADP_ = (l/100 + 0.16 × m/100 + 0.0256 × n/100 + 0.0041 × p/100 + 0.0007 × q/100 + 0.0001 × r/100 − 0.32)^2^ + (0.84 × m/100 + 0.2688 × n/100 + 0.0645 × p/100 + 0.0138 × q/100 + 0.0028 × r/100 + 0.0005 × s/100 − 0.37)^2^ + (0.7056 × n/100 + 0.3387 × p/100 + 0.1084 × q/100 + 0.0289 × r/100 + 0.0069 × s/100 − 0.23)^2^ + (0.5927 × p/100 + 0.3793 × q/100 + 0.1517 × r/100 + 0.0486 × s/100 – 0.08)^2^ + (0.4978 × q/100 + 0.3983 × r/100 + 0.1912 × s/100)^2^ + (0.4182 × r/100 + 0.4015 × s/100)^2^ + (0.3513 × s/100)^2^.

H_ATPγS_ = (l/100 + 0.16 × m/100 + 0.0256 × n/100 + 0.0041 × p/100 + 0.0007 × q/100 + 0.0001 × r/100 − 0.29)^2^ + (0.84 × m/100 + 0.2688 × n/100 + 0.0645 × p/100 + 0.0138 × q/100 + 0.0028 × r/100 + 0.0005 × s/100 − 0.35)^2^ + (0.7056 × n/100 + 0.3387 × p/100 + 0.1084 × q/100 + 0.0289 × r/100 + 0.0069 × s/100 − 0.24)^2^ + (0.5927 × p/100 + 0.3793 × q/100 + 0.1517 × r/100 + 0.0486 × s/100 – 0.10)^2^ + (0.4978 × q/100 + 0.3983 × r/100 + 0.1912 × s/100 – 0.02)^2^ + (0.4182 × r/100 + 0.4015 × s/100)^2^ + (0.3513 × s/100)^2^.

H_ADP and ATPγS_ = (l/100 + 0.16 × m/100 + 0.0256 × n/100 + 0.0041 × p/100 + 0.0007 × q/100 + 0.0001 × r/100 − 0.19)^2^ + (0.84 × m/100 + 0.2688 × n/100 + 0.0645 × p/100 + 0.0138 × q/100 + 0.0028 × r/100 + 0.0005 × s/100 − 0.35)^2^ + (0.7056 × n/100 + 0.3387 × p/100 + 0.1084 × q/100 + 0.0289 × r/100 + 0.0069 × s/100 − 0.30)^2^ + (0.5927 × p/100 + 0.3793 × q/100 + 0.1517 × r/100 + 0.0486 × s/100 – 0.13)^2^ + (0.4978 × q/100 + 0.3983 × r/100 + 0.1912 × s/100 – 0.02)^2^ + (0.4182 × r/100 + 0.4015 × s/100 – 0.01)^2^ + (0.3513 × s/100)^2^.

l, m, n, p, q, r, and s indicate percentages of 0, 1, 2, 3, 4, 5, and 6 RuvB dimers binding to a RuvA-Holliday junction DNA. The l, m, n, p, q, r, and s satisfied the following conditions.


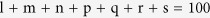


l, m, n, p, q, r, and s were nonnegative integers.

The inequalities as below were not allowed.


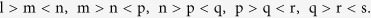


## Additional Information

**How to cite this article**: Iwasa, T. *et al.* Synergistic effect of ATP for RuvA—RuvB—Holliday junction DNA complex formation. *Sci. Rep.* **5**, 18177; doi: 10.1038/srep18177 (2015).

## Supplementary Material

Supplementary Movie S1

Supplementary Tables S1-S3

## Figures and Tables

**Table 1:** Distribution of the number of RuvBs binding to a RuvA–Holliday junction DNA from the fitted data calculated from Supplementary Table S1.

	**No Nucleotide**	**ADP**	**ATPgS**	**ADP + ATPgS**
0 RuvBs	23%	10%	8%	2%
1 RuvBs	37%	10%	12%	5%
2 RuvBs	40%	21%	18%	8%
3 RuvBs	0%	30%	19%	31%
4 RuvBs	0%	29%	31%	37%
5 RuvBs	0%	0%	12%	14%
6 RuvBs	0%	0%	0%	3%

**Table 2:** Distribution of the number of RuvBs binding to a RuvA–Holliday junction DNA from the fitted data calculated from Supplementary Table S2.

	**No Nucleotide**	**ADP**	**ATPgS**	**ADP + ATPgS**
0 RuvB dimers	35%	11%	11%	3%
1 RuvB dimers	65%	48%	36%	24%
2 RuvB dimers	0%	41%	53%	70%
3 RuvB dimers	0%	0%	0%	3%
4 RuvB dimers	0%	0%	0%	0%
5 RuvB dimers	0%	0%	0%	0%
6 RuvB dimers	0%	0%	0%	0%

**Table 3:** Distribution of the number of RuvBs binding to a RuvA–Holliday junction DNA from the fitted data calculated from Supplementary Table S3.

	**No Nucleotide**	**ADP**	**ATPgS**	**ADP** **+** **ATPgS**
0 RuvB dimers	52%	26%	23%	14%
1 RuvB dimers	38%	35%	32%	29%
2 RuvB dimers	10%	26%	27%	33%
3 RuvB dimers	0%	13%	14%	19%
4 RuvB dimers	0%	0%	4%	4%
5 RuvB dimers	0%	0%	0%	1%
6 RuvB dimers	0%	0%	0%	0%
