# Supplementary material for: Synergistic effect of ATP for RuvA–RuvB–Holliday junction DNA complex formation
Source: Sci Rep. 2015 Dec 14;5:18177. doi: 10.1038/srep18177 (PMC4677358; doi:10.1038/srep18177)
Supplement: Supplementary Tables S1-S3 [file srep18177-s2.doc]

**Supplementary Information for**

**Synergistic effect of ATP for RuvA–RuvB–Holliday junction DNA complex formation**

Takuma Iwasa, Yong-Woon Han, Ryo Hiramatsu, Hiroaki Yokota, Kimiko Nakao, Ryuji Yokokawa, Teruo Ono and Yoshie Harada

**Supplementary information Contents**

Supplementary Table S1. Ratio of Cy5-RuvB calculated by the binominal distribution.

Supplementary Table S2. Ratio of Cy5-RuvB calculated by the binominal distribution.

Supplementary Table S3. Ratio of Cy5-RuvB dimer calculated by the binominal distribution.

Supplementary Movie S1. Observation of RuvA–Cy5-RuvB–Cy3-Holliday junction DNA complex in the nanoholes.

Supplementary Table S1

Ratio of Cy5-RuvB calculated by the binominal distribution

|  | | Total Number of RuvB (Cy5-RuvB and RuvB-S39C) | | | | | | |
| --- | --- | --- | --- | --- | --- | --- | --- | --- |
|  |  | 0 | 1 | 2 | 3 | 4 | 5 | 6 |
| Number of Cy5-RuvB | 0 | 1 | 0.58 | 0.3364 | 0.1951 | 0.1132 | 0.0656 | 0.0381 |
|  | 1 | 0 | 0.42 | 0.4872 | 0.4239 | 0.3278 | 0.2377 | 0.1654 |
|  | 2 | 0 | 0 | 0.1764 | 0.3069 | 0.3560 | 0.3442 | 0.2994 |
|  | 3 | 0 | 0 | 0 | 0.0741 | 0.1719 | 0.2492 | 0.2891 |
|  | 4 | 0 | 0 | 0 | 0 | 0.0311 | 0.0902 | 0.1570 |
|  | 5 | 0 | 0 | 0 | 0 | 0 | 0.0131 | 0.0455 |
|  | 6 | 0 | 0 | 0 | 0 | 0 | 0 | 0.0055 |

The calculation of the binominal distribution was based on the assumption that the RuvB protomer is a monomer.

Supplementary Table S2

Ratio of Cy5-RuvB calculated by the binominal distribution

|  | | Total Number of RuvB (Cy5-RuvB and RuvB-S39C) | | | |
| --- | --- | --- | --- | --- | --- |
|  |  | 0 | 2 | 4 | 6 |
| Number of Cy5-RuvB | 0 | 1 | 0.3364 | 0.1132 | 0.0381 |
|  | 1 | 0 | 0.4872 | 0.3278 | 0.1654 |
|  | 2 | 0 | 0.1764 | 0.3560 | 0.2994 |
|  | 3 | 0 | 0 | 0.1719 | 0.2891 |
|  | 4 | 0 | 0 | 0.0311 | 0.1570 |
|  | 5 | 0 | 0 | 0 | 0.0455 |
|  | 6 | 0 | 0 | 0 | 0.0055 |

The calculation of the binominal distribution was based on the assumption that the RuvB protomer is a dimer and Cy5-RuvB is distributed in RuvB dimers at random.

Supplementary Table S3

Ratio of Cy5-RuvB dimer calculated by the binominal distribution

|  | | Total Number of RuvB dimer (Cy5-RuvB and RuvB-S39C) | | | | | | |
| --- | --- | --- | --- | --- | --- | --- | --- | --- |
|  |  | 0 | 1 | 2 | 3 | 4 | 5 | 6 |
| Number of Cy5-RuvB | 0 | 1 | 0.16 | 0.0256 | 0.0041 | 0.0007 | 0.0001 | 0 |
|  | 1 | 0 | 0.84 | 0.2688 | 0.0645 | 0.0138 | 0.0028 | 0.0005 |
|  | 2 | 0 | 0 | 0.7056 | 0.3387 | 0.1084 | 0.0289 | 0.0069 |
|  | 3 | 0 | 0 | 0 | 0.5927 | 0.3793 | 0.1517 | 0.0486 |
|  | 4 | 0 | 0 | 0 | 0 | 0.4978 | 0.3983 | 0.1912 |
|  | 5 | 0 | 0 | 0 | 0 | 0 | 0.4182 | 0.4015 |
|  | 6 | 0 | 0 | 0 | 0 | 0 | 0 | 0.3513 |

The calculation of the binominal distribution was based on an assumption that the RuvB protomer is a dimer and all Cy5-RuvB dimers contain a single Cy5-RuvB.

Supplementary Movie S1. Observation of RuvA–Cy5-RuvB–Cy3-Holliday junction DNA complex in the nanoholes. This movie shows Cy5-RuvBs binding to RuvA–Cy3-Holliday junction DNA complex. Top panel shows fluorescent signal from Cy3-Holliday junction DNA and bottom panel shows fluorescent signal from Cy5-RuvB. The area displayed is 24 μm × 24 μm.
